# Foreign Body Aspiration Mimicking an Endobronchial Neoplasm: A Case Report and Review of the Literature

**DOI:** 10.7759/cureus.36105

**Published:** 2023-03-13

**Authors:** Kush Purohit, Samuel Grandfield, Ankit Dhamija, Almas Abbasi

**Affiliations:** 1 Radiology, Stony Brook University Hospital, Stony Brook, USA; 2 Cardiothoracic Surgery, Robotic Thoracic Surgery, Stony Brook University Hospital, Stony Brook, USA; 3 Radiology, Abdominal and Thoracic Imaging, Stony Brook University Hospital, Stony Brook, USA

**Keywords:** bronchoscopy, low dose chest ct screening, squamous metaplasia, fdg pet-ct, tracheobronchial foreign body aspiration

## Abstract

Foreign body aspiration (FBA) is infrequently encountered in the adult population, with major risk factors including advancing age, intoxication, and disorders of the central nervous system. Here, we present a case of FBA in an adult undergoing routine lung cancer screening to review imaging findings and highlight potential pitfalls for the practicing radiologist. A low-dose chest computed tomography (CT) scan was performed for lung cancer screening in a 57-year-old male with a one-month history of worsening dyspnea and cough. An endobronchial lesion was identified in the right bronchus intermedius. A follow-up 18F-fluorodeoxyglucose positron emission tomography-computed tomography (18F-FDG PET-CT) revealed hypermetabolic activity in the region of interest, raising concern for malignancy. Bronchoscopy was performed, revealing a nodular mass adjacent to a foreign body in the bronchus intermedius. Histopathologic analysis of the tissue sample revealed the presence of an aspirated foreign body with squamous metaplasia of the respiratory epithelium. Adult FBA is an uncommon clinical entity that may be incidentally observed on a screening chest CT. Relevant multimodality imaging findings are discussed here, along with a review of the accompanying pathologic changes seen with chronic airway impaction.

## Introduction

Foreign body aspiration (FBA) is typically encountered in the pediatric population [1]. Given its relative infrequency and varied clinical presentation in adults, radiologic imaging is of critical importance in confirming suspected FBA in older populations. Rarely, FBA may be incidentally discovered on radiologic imaging and pose a challenge to the diagnostic radiologist, particularly in the absence of an associated clinical history. Here, we present a unique case of FBA incidentally identified on routine screening to describe key imaging features and explore potential diagnostic pitfalls.

## Case presentation

Initial presentation 

A 57-year-old man with a history of HIV, hypertension, chronic obstructive pulmonary disease (COPD), and a 25-pack-year active smoking history presented for an annual lung cancer screening appointment. The patient reported a recent one-month history of shortness of breath and a non-productive cough. He also described the feeling that "no air was going into the right side of my lungs". He denied hemoptysis, weight loss, fatigue, fevers, chills, night sweats, chest pain, or palpitations.

On physical exam, the patient’s vital signs revealed hypoxia with an oxygen saturation (SpO2) of 93% and mild bilateral rhonchi on chest auscultation.

Imaging

**Figure 1:**
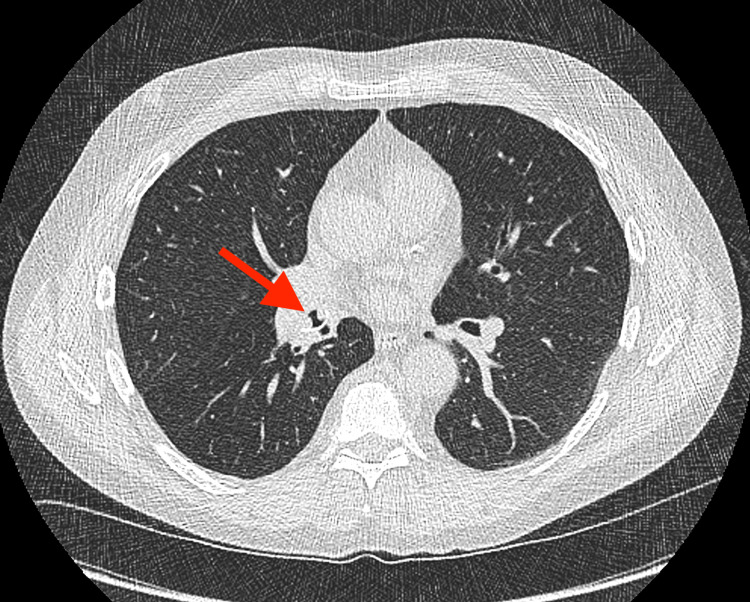
An axial image of a low-dose chest CT without intravenous contrast demonstrating a nodular, partially calcified endobronchial lesion in the right bronchus intermedius (red arrow).

**Figure 2:**
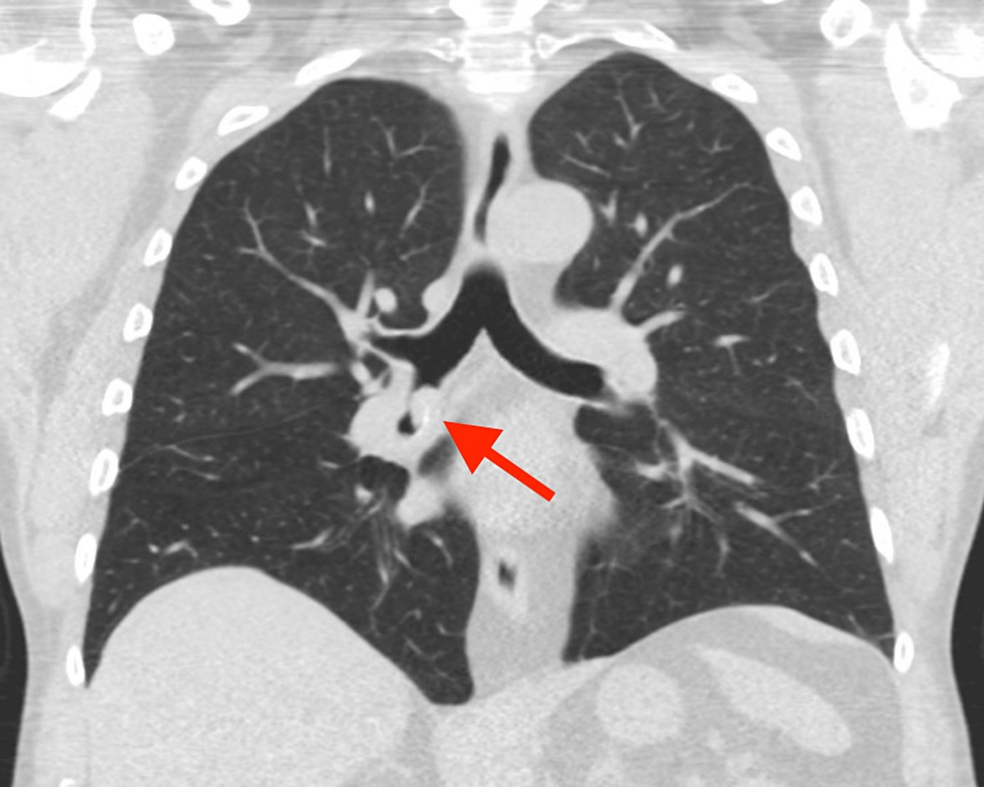
A coronal image of the same low-dose chest CT revealing the endobronchial lesion along the inferior aspect of the bronchus intermedius lumen (red arrow).

A low-dose screening computed tomography (CT) of the chest was performed as a part of lung cancer screening and demonstrated a 1.2 cm nodular endobronchial lesion in the distal bronchus intermedius (Figures 1, 2). The lesion contained multiple internal punctate calcific foci and was adherent to the walls of the bronchus intermedius just proximal to its bifurcation into the right middle and lower lobe bronchi. No additional endobronchial lesions, pulmonary nodules, or enlarged lymph nodes were identified.

**Figure 3:**
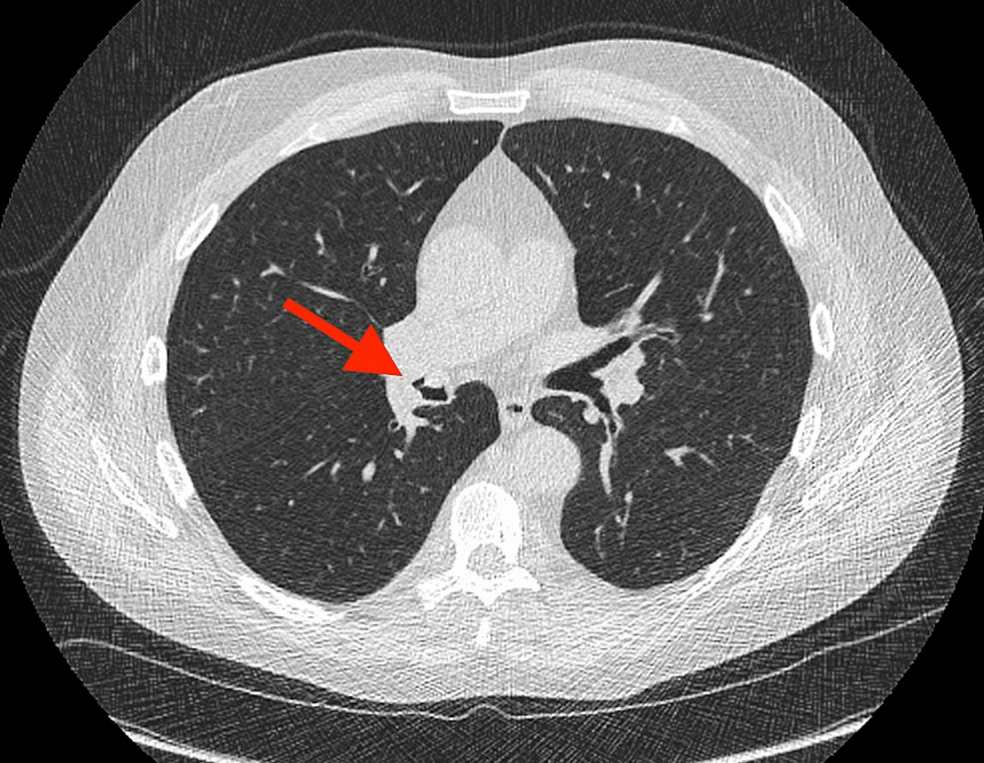
An axial image of the low-dose chest CT without intravenous contrast performed two years prior, with the endobronchial lesion again seen within the bronchus intermedius (red arrow), apparently smaller in size in comparison to the more recent study.

**Figure 4:**
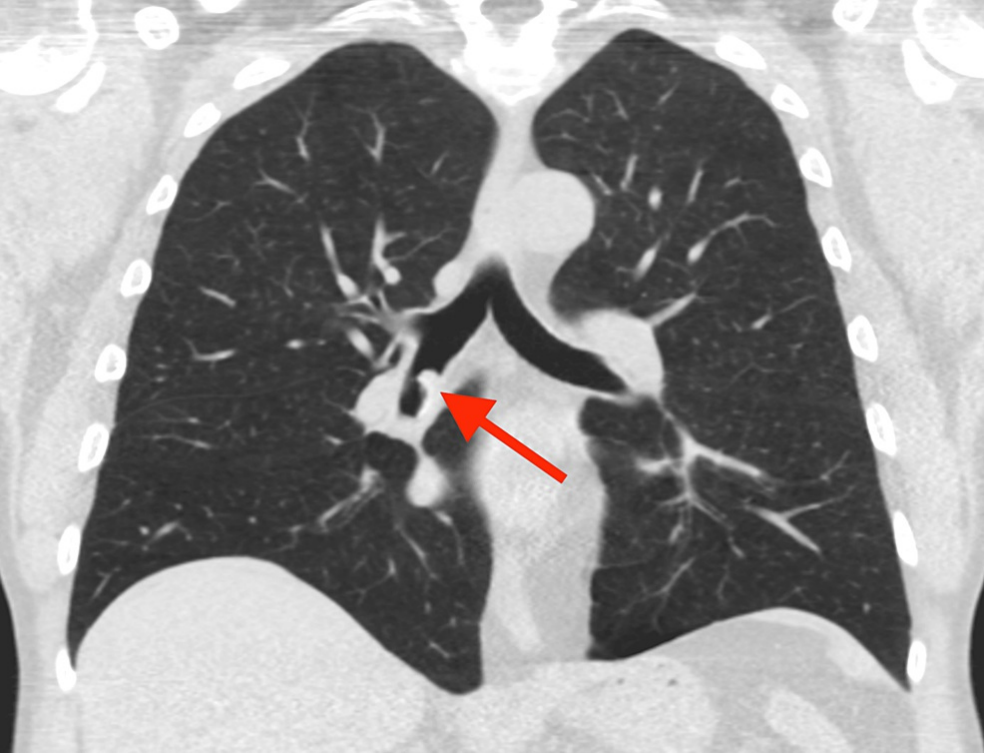
A coronal image of the same prior exam shows the partially calcified lesion in an unchanged position within the bronchus intermedius (red arrow).

A retrospective review of prior imaging was performed, and the endobronchial lesion was identified on a low-dose chest CT performed two years prior (Figures 3, 4). The current exam suggested slight interval growth of the lesion from 0.9 cm to 1.2 cm with a similar partially calcified, irregular nodular appearance.

**Figure 5:**
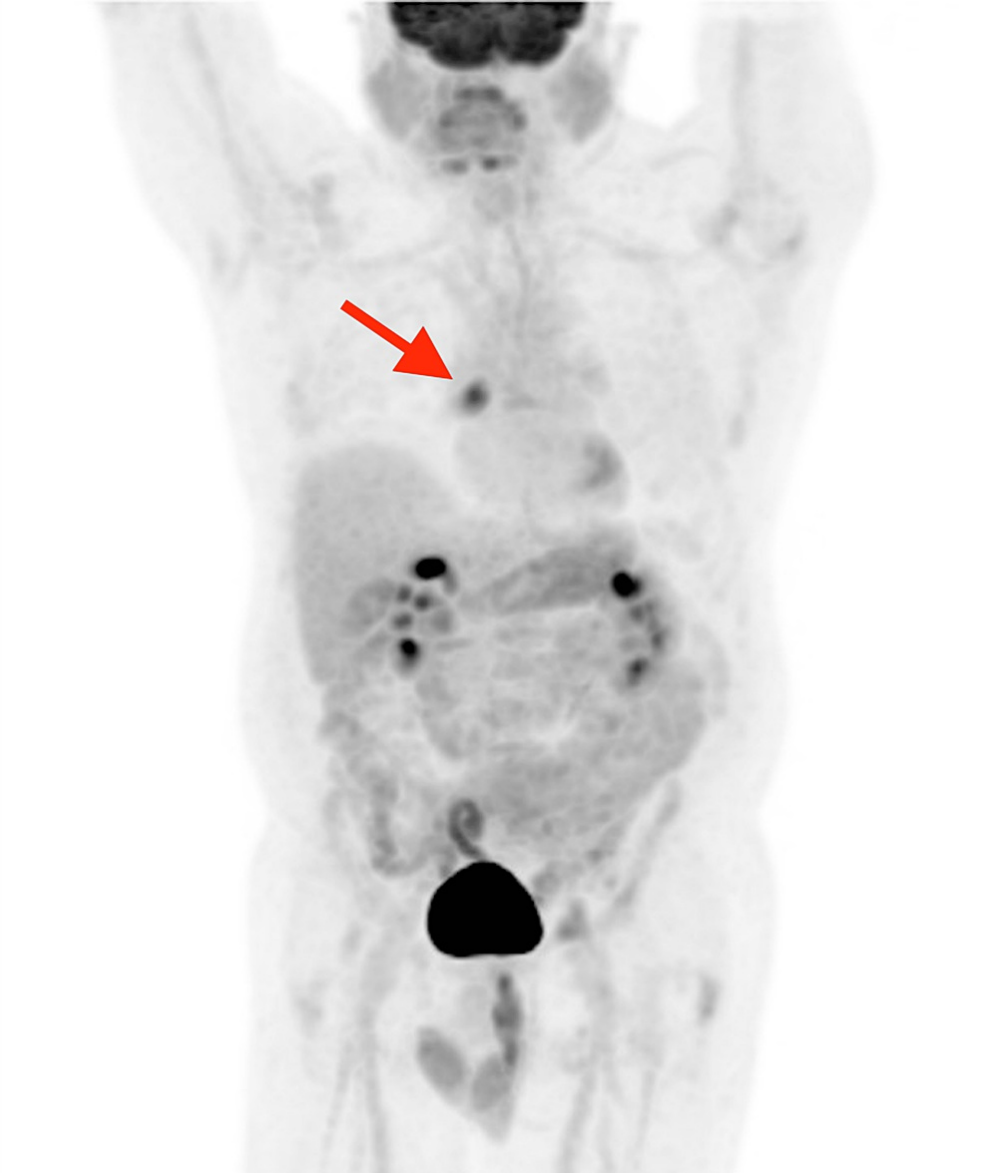
A coronal maximum intensity projection image of the FDG PET-CT revealing a hypermetabolic focus in the right hemithorax (red arrow).

**Figure 6:**
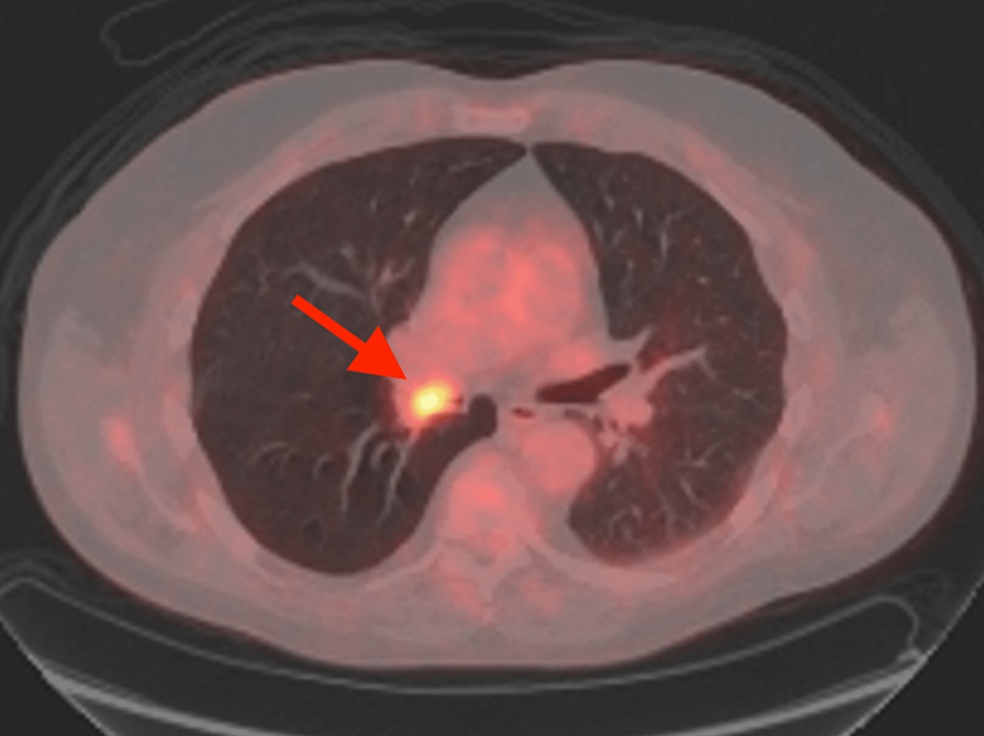
An axial fused image of the same study demonstrating the hypermetabolic region corresponding to the endobronchial lesion in the right bronchus intermedius (red arrow).

A hybrid 18F-fluorodeoxyglucose positron emission tomography-computed tomography (18F-FDG PET-CT) was performed to evaluate the metabolic activity of the lesion. A 1.2 x 1.7 cm hypermetabolic focus with a standardized uptake value (SUVmax) of 7.2 was identified in the right hemithorax, corresponding to an endobronchial soft tissue nodule in the right bronchus intermedius (Figures 5, 6). Findings raised concern for a slowly growing endobronchial malignancy in this patient at high risk for malignancy. No additional hypermetabolic foci or FDG-avid lymph nodes were visualized.

Bronchoscopy

**Figure 7:**
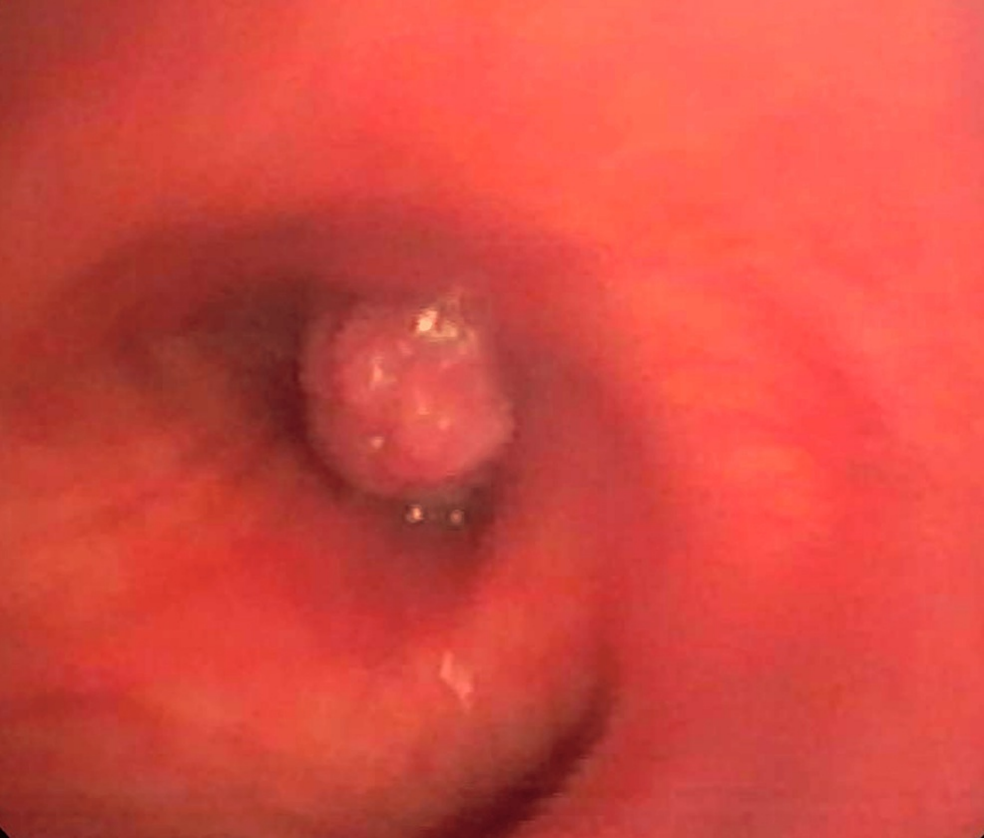
An intraoperative bronchoscopic image demonstrating an ovoid, flesh-colored soft tissue nodule at the proximal bronchus intermedius projecting into the airway lumen. Biopsies of the nodule were obtained and sent for histopathologic analysis.

**Figure 8:**
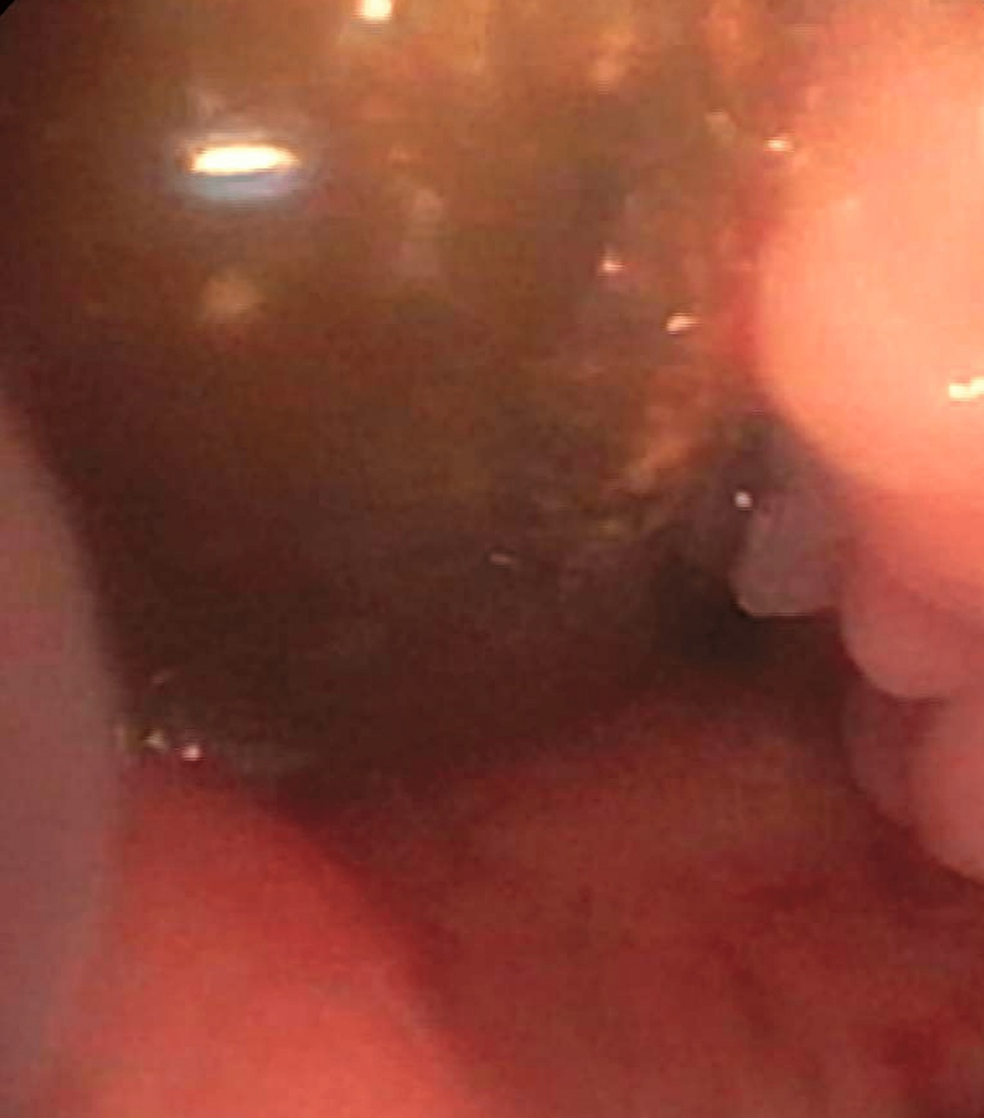
A bronchoscopic image of a brown, curved foreign body was identified distal to the soft tissue nodule, just proximal to the bifurcation of the bronchus intermedius.

**Figure 9:**
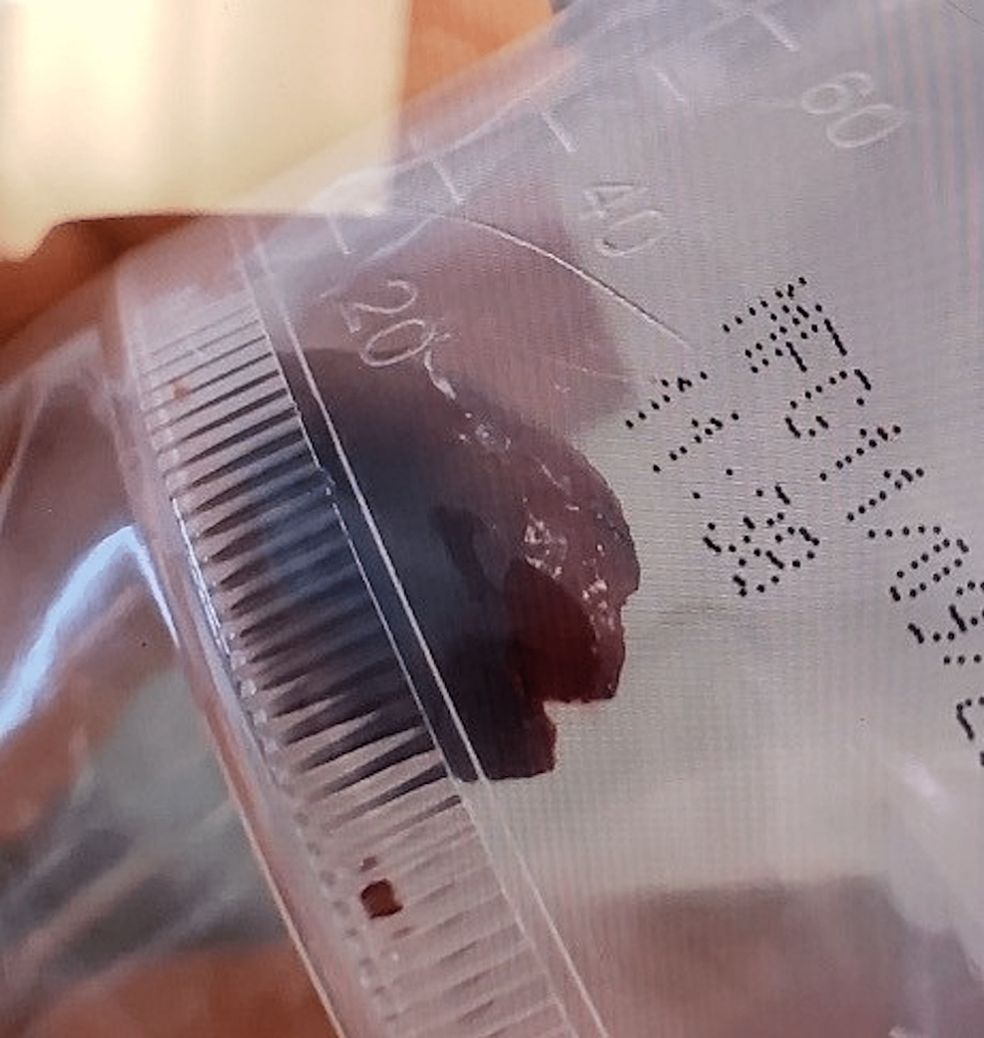
The endobronchial foreign body in a specimen container.

Flexible bronchoscopy with endobronchial ultrasound was then performed, revealing an ovoid soft tissue mass within the bronchus intermedius measuring up to 0.8 cm (Figures 7-9). Biopsies of the lesion and surrounding mucosa were obtained and sent for histopathologic analysis. Distal to the mass, a curved, reddish-brown foreign body was identified just proximal to the bifurcation into the right middle and lower lobe bronchi. The foreign body was successfully removed using alligator forceps. Endobronchial ultrasound was performed at the paratracheal and subcarinal areas without evidence of lymphadenopathy.

 

Pathology

Histopathologic analysis of the endobronchial mass revealed benign squamous metaplasia and hyperplasia with stromal fibrosis. Tissue samples of the surrounding mucosa demonstrated benign squamous metaplasia with mild chronic inflammatory changes. No malignant cells were identified. The curved, reddish-brown foreign body removed from the bronchus intermedius was grossly examined and identified as a pistachio shell. 

Clinical follow-up

**Figure 10:**
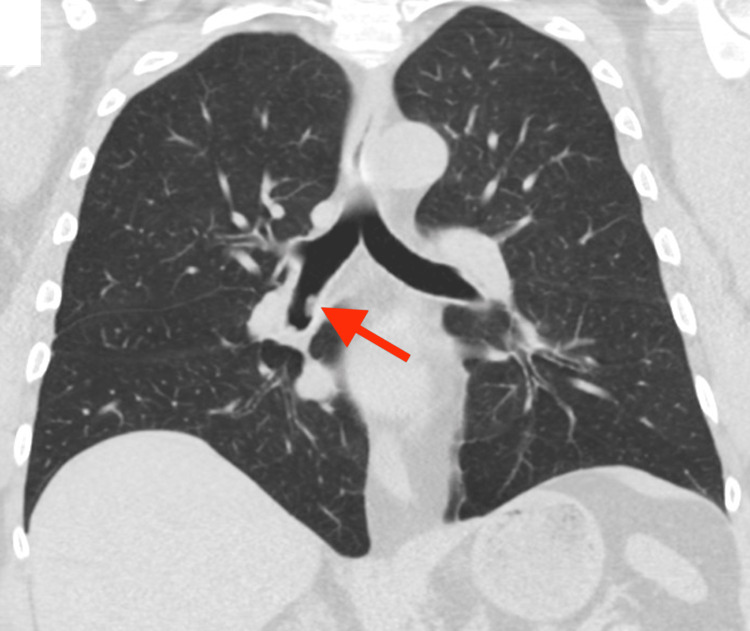
A coronal image of the follow-up chest CT without intravenous contrast demonstrating the interval removal of the endobronchial foreign body with a subcentimeter-sized residual soft nodule within the bronchus intermedius (red arrow).

On follow-up examination, the patient reported "feeling like a new man". He stated his cough and shortness of breath resolved following the bronchoscopic intervention. A follow-up CT of the chest was obtained, which revealed the interval removal of the right bronchus intermedius lesion seen in prior studies. A small, irregular soft tissue nodule measuring up to 0.3 cm was seen, suggesting residual inflammatory change at the procedural site (Figure 10).

## Discussion

Foreign body aspiration (FBA) is a relatively uncommon phenomenon in the adult population. Indeed, a large recent retrospective study by Ulas et al. determined that only 11.7% of FBA cases were seen in adult patients (>18 years old) [1]. Aspiration in the pediatric population, however, is the fourth leading cause of accidental mortality in patients under the age of three years [2]. Despite its relative infrequency, the incidence of adult deaths due to FBA increases with advancing age beyond the sixth decade [3]. Additional risk factors in the adult population include trauma, altered mentation as a result of sedative or alcohol use, or primary neurologic disorders such as stroke or seizure disorders [4,5].

The most common symptoms associated with FBA in adults include a sudden onset of choking, coughing, dyspnea, fever, hemoptysis, and wheezing [1,6,7]. The type of aspirated object varies depending on the medical environment and cultural practices of the patient population [5,8]. For instance, aspiration of teeth, animal bones, nuts, seeds, beans, and cones comprised over two-thirds of adult cases in a Korean population, while aspiration of needles, metallic objects, and food particles was responsible for over 70% of cases in a single-center study performed in Turkey [1,6]. Interestingly, pistachios have been implicated in a number of reported cases across the globe. The right bronchus intermedius is the most common site for airway impaction with adult FBA, which is likely secondary to its relatively vertical configuration. The left and right mainstem bronchi are the second and third most common sites for aspiration, respectively.

The diagnosis of FBA is typically achieved with a combination of detailed history-taking, diagnostic imaging, and bronchoscopy. Standard chest radiography is the initial imaging modality of choice in cases of suspected aspiration. However, the variable radiopacity of aspirated objects results in the inconsistent sensitivity reported in the literature, ranging from 8% to 80% [9]. Secondary signs of FBA on radiography such as obstructive atelectasis, focal post-obstructive consolidation, and air trapping can be useful to reinforce clinical suspicion and direct further imaging or intervention [6,10].

A chest CT may be performed following initial radiography to identify radiolucent foreign bodies not seen on X-ray as well as to provide anatomic detail for procedural planning. Interestingly, the patient in the case presented here underwent routine annual cancer screening CT for lung cancer, which is when the aspirated foreign body was incidentally discovered. Jang et al. found that CT studies reliably identify tracheobronchial foreign bodies when compared to standard radiography, with greater than 60% sensitivity [6]. When a foreign body is not readily identified, secondary findings such as airway obstruction, mucus plugging, bronchial wall thickening, air trapping, post-obstructive atelectasis, or pneumonia are suggestive of FBA and warrant further bronchoscopic evaluation following CT.

Radiologic evaluation for patients with suspected FBA does not typically involve nuclear imaging. However, in the case presented here, FDG PET-CT was performed due to a suspicion of malignancy. A single hypermetabolic focus was identified in the bronchus intermedius, which raised further suspicion for neoplasm in this patient within the "intermediate risk" category when risk-stratified using the PLCOM2012 model described by Tammemagi et al [11]. An increase in FDG uptake has been documented in cases of chronic indwelling foreign bodies, which have been shown to produce chronic airway irritation and inflammation [12-14]. This is likely due to the activation of inflammatory cells, which can overexpress glucose receptors and therefore facilitate increased FDG uptake [15]. The vast majority of the literature describes hypermetabolic activity secondary to the iatrogenic placement of foreign bodies in post-surgical patients with known malignancy histories (including nonabsorbable suture material, polysaccharide-based hemostatic agents, or breast implant material) [12-14,16]. Our case demonstrates a case of FBA masquerading as an occult malignancy on PET-CT in a surgically naïve patient without a history of malignancy.

Histopathologic analysis of the tissue samples obtained in our case similarly demonstrated sequela of chronic inflammatory changes; these findings include lymphocyte and plasma cell infiltration, fibrosis, and the formation of foreign body giant cells and granulomas [12]. These changes were seen in the samples obtained in our case, including in the mass-like nodule adjacent to the aspirated pistachio shell, which also revealed squamous metaplasia.

Respiratory pseudostratified columnar epithelium normally lines the conducting airways of the respiratory tract. Metaplastic change to the squamous epithelium is classically seen as secondary to the toxic insult of the airways as a precursor to the development of squamous cell carcinoma. This phenomenon has been widely documented to occur in the bronchial tissue of smokers, with squamous metaplastic cells expressing increased levels of carcinoembryonic antigen [17]. Additionally, chronic inflammation due to retained oral chaff debris in rat feed has been observed to induce squamous metaplasia with progression to squamous cell carcinoma [18]. This is consistent with our case, in which squamous metaplasia was seen in addition to the expected chronic inflammatory changes. As both chronic smoking and FBA have been shown to elicit squamous metaplasia, it is, therefore, unclear whether the aspirated pistachio shell or smoking history was chiefly responsible for the cellular changes.

Once FBA is identified or suspected with corroborative clinical history and imaging findings, bronchoscopy is the next step in management for foreign body removal. Flexible bronchoscopy is the favored procedure in the adult population as it produces less patient discomfort, may be performed under light sedation, and uniquely allows access to the distal and peripheral airways [19]. However, as a larger working port is required for flexible bronchoscopy, rigid bronchoscopy has historically been favored for foreign body removal in the pediatric population and also allows superior airway control. In patients with known aspiration risk factors, prevention strategies should be employed, such as upright positioning during meals, mealtime supervision, and routine oral care [20].

## Conclusions

FBA is relatively uncommon in the adult population, particularly in the absence of known risk factors such as trauma, intoxication, or primary neurologic disorders. Here, we present a unique case of an aspirated foreign body incidentally identified on low-dose chest CT to highlight a potential pitfall for the interpreting radiologist. As updated recommendations for lung cancer screening result in a broader population now eligible for low-dose chest CT, it is important for radiologists to recognize alternative entities that may mimic malignancy on traditional and hybrid imaging modalities. This case also highlights the context-dependent nature of diagnostic radiology, as the search for occult malignancy on this screening CT proved to obfuscate the correct diagnosis.
